# Metabolism and pharmacokinetics of morphine in neonates: A review

**DOI:** 10.6061/clinics/2016(08)11

**Published:** 2016-08

**Authors:** Gian Maria Pacifici

**Affiliations:** University of Pisa, Neurosciences Medical Scholl, via Roma 55, Pisa 56126, Italy

**Keywords:** Metabolism, Morphine, Neonate, Pharmacokinetics

## Abstract

Morphine is an agonist of the µ and k receptors, whose activation results in analgesia. Morphine-like agonists act through the µ opioid receptors to cause pain relief, sedation, euphoria and respiratory depression. Morphine is glucuronidated and sulfated at positions 3 and 6; the plasma concentration ratios correlate positively with birth weight, which probably reflects increased liver weight with increasing birth weight. Moreover, morphine clearance correlates positively with gestational age and birth weight. Steady-state morphine plasma concentrations are achieved after 24-48 hours of infusion, but the glucuronide metabolite plasma concentrations do not reach steady state before 60 hours. The morphine-3-glucuronide metabolite has lower clearance, a shorter half-life and a smaller distribution volume compared with the morphine-6 metabolite, which is the most active morphine-like agonist. Ordinary doses cause constipation, urinary retention and respiratory depression. Neonatal pain relief may require a blood level of approximately 120 ng/ml, whereas lower levels (20-40 ng/ml) seem adequate for children. A bibliographic search was performed using the PubMed database and the keywords “morphine metabolism neonate” and “morphine pharmacokinetics neonate”. The initial and final cutoff points were January 1990 and September 2015, respectively. The results indicate that morphine is extensively glucuronidated and sulfated at positions 3 and 6, and that the glucuronidation rate is lower in younger neonates compared with older infants. Although much is known about morphine in neonates, further research will be required to ensure that recommended therapeutic doses for analgesia in neonates are evidence based.

## INTRODUCTION

Morphine is an agonist of both the µ and the k receptors, and activation of these receptors results in analgesia. Morphine-like agonists mediate their effects via the µ opioid receptors to cause pain relief, sedation, euphoria and respiratory depression. Morphine blocks the transmission of nociceptive signals, activates signaling by pain-modulating neurons to the spinal cord, and inhibits transmission from primary afferent nociceptors to dorsal horn sensory projection cells 1. With increasing doses, the degree of analgesia increases until an anesthetic level is reached. The onset of morphine-induced analgesia after intravenous administration is relatively slow (6-30 min), partly because of its limited lipid solubility and its slow rate of penetration through the blood-brain barrier. Additionally, morphine undergoes significant first-pass metabolism; thus, oral doses must be six-fold greater than parenteral doses to achieve the same degree of analgesia 2. However, the short-term elimination half-life of 3-4 hours in adults limits the duration of analgesia.

Morphine, which is the main alkaloid of opium, was first obtained from poppy heads in 1805 3. The half-life in preterm infants is 6-12 hours but is very variable and is inversely related to gestational age at birth. Certain tissue accumulation occurs with sustained use, with a distribution volume of approximately 2 l/kg. Elimination is more rapid in infants older than 2 months, with a half-life of approximately 1 hour in 1- to 6-year-old children. Neonatal pain relief may require blood levels of approximately 120 ng/ml, although overdose effects start to appear at levels exceeding 300 ng/ml. Lower levels, or approximately 20-40 ng/ml, appear to be adequate in older children 3. The higher levels required in the newborn may reflect drug receptor differences and low metabolization to morphine-6-glucuronide, which is essentially the active metabolite. Tolerance may develop with prolonged treatment, and withdrawal symptoms can also occur. Addiction has not been observed with neonatal use for pain relief, but there are concerns that long-term opiate exposure may affect visual function. Moreover, neonatal abstinence due to opiate withdrawal produces sleep/wake abnormalities, feeding difficulties, weight loss and seizures. An analysis of the management of morphine weaning has recently been presented elsewhere 4.

Morphine is frequently used in infants undergoing therapeutic hypothermia. Morphine’s affinity for the µ opioid receptors is reduced in hypothermia, rendering it less effective, at least in the early stages; however, because the clearance of morphine is lower in the very young newborn, accumulation may occur if higher doses are used. Accordingly, the dose of morphine should be controlled and reduced if the infant is adequately sedated after 24-48 hours, lessening the risk of accumulation and toxicity 3. In addition, naloxone should be readily available to reverse marked hypotension and bradycardia. Paralytic ileus, delayed gastric empting, urine retention and tolerance may develop after prolonged use; therefore, weaning should be performed slowly.

This review covers the results reported on the subject over the past 25 years. In particular, this review puts into perspective the most significant progress made in terms of the metabolic pathways and the pharmacodynamic properties of morphine in human neonates and infants. The most controversial point related to morphine use in the perinatal period is the dosage to be employed to both ensure adequate action and minimize safety concerns. We believe that this review offers an adequate framework within which morphine can be used.

## BIBLIOGRAPHIC SEARCH

A bibliographic search was performed using the PubMed database as the search engine. January 1990 and September 2015 were the initial and final cutoff points, respectively. Three other specific references were also inserted for historical clarification. When the keywords “morphine metabolism neonate” and “morphine pharmacokinetics neonate” were used, a total of 69 original research papers were located. The abstracts and subsequently the full articles were analyzed, and ultimately, a total of 24 articles were selected for analysis. In addition, the books *Neonatal Formulary* 3 and *NeoFax*, by Young and Mangum, were consulted 5. Methadone, other hypnoanalgesic drugs and central stimulants will be briefly referred to wherever appropriate, and alternative sedative agents have been recently reviewed 6,7.

## RESULTS

### Metabolism of Morphine

Table 1 displays data relating to ten original research reports mainly centered on the metabolism of morphine. These reports are presented in chronological sequence as a historical survey of the evolution of the topic.

Choonara et al. 8 studied the sulfation of morphine in 9 children and 7 preterm neonates. All of the neonates and 3 children had detectable concentrations of morphine-3-sulfate in the urine, but none had a detectable concentrations of morphine-6-sulfate in the urine. Additionally, none of the children had a detectable concentration of morphine-3-sulfate in the plasma. Oxidation and sulfation are thought to be minor pathways of morphine metabolism, so the ratio of morphine-3-sulfate to morphine can be used as an index of sulfation. This ratio, however, is very low, even in the neonatal period, indicating that the contribution of sulfation to the metabolism of morphine is minimal. In another study, Choonara et al. 9 demonstrated that full-term neonates and older infants have a well-developed mechanism for the glucuronidation of morphine at both position 3 and position 6. In all cases, the plasma concentrations of morphine-3-glucuronide were higher than those of morphine and the plasma concentrations of morphine-6-glucuronide were higher than those of morphine in 7 of the 10 infants in whom morphine-6-glucuronide was detectable. The ratio of morphine-3-glucuronide to morphine in the plasma was described as a useful index of glucuronidation in the presence of normal renal function. Bhat et al. 10 examined the manner in which morphine is metabolized in 16 acutely ill very premature (25-32 weeks gestational age) infants. Morphine was detected in the plasma at 4 hours after dosing in 13 patients and at 24 hours in 12 patients, and large amounts of unmetabolized morphine were found in the urine at 4 and 24 hours. Nearly two thirds of acutely ill preterm infants born at less than 32 weeks gestational age exhibited metabolism of morphine to morphine-3-glucuronide, which is not an analgesic and to morphine-6-glucuronide, which is a highly potent analgesic and sedative. McRorie et al. 11 investigated the clearance of morphine and morphine sulfate in 49 children aged 1 day to 2.5 years with normal renal and hepatic function and stable hemodynamics. Morphine clearance reached the adult value between the second week and the sixth month of life. The formation clearance of morphine glucuronides was correlated with age, whereas the formation clearance of morphine sulfate was independent of age. In another study, Hartley et al. 12 studied morphine pharmacokinetics in 17 premature neonates (26-34 weeks of gestation) after intravenous infusion during the first 24 hours of life. Infants received either a standard loading dose of morphine (100 µg/kg/h) or a high dose (200 µg/kg/h), both followed by a maintenance infusion. The mean plasma concentrations of morphine after 2 and 24 hours were respectively 99±12.9 and 96.4±3.2 for the standard-dose regimen and 184±37.7 and 319±71.2 for the high-dose regimen. Morphine-3-glucuronide plasma concentrations achieved approximately 20% and 80% of morphine values at 2 and 24 hours, respectively. Morphine-6-glucuronide could not be detected at 2 hours but attained 20-25% of the morphine plasma concentration by 24 hours. Furthermore, Hartley et al. 13 provided the first clear-cut *in vivo* indication of the differential development of uridine diphosphate glucuronosyltransferase in neonates. In premature neonates (25-34 weeks of gestation), only morphine and morphine-3-glucuronide were detected in plasma obtained over the first 24 hours of life; morphine-6-glucuronide could only be quantified after 24 hours. The concentrations of both active metabolites increased significantly with increasing birth weight. However, the ratio of the morphine-6 concentration to the morphine-3-glucuronide concentration decreased with increasing birth weight (and gestational age). High plasma concentrations of morphine appeared to be well tolerated. Lynn et al. 14 analyzed morphine clearance values in 35 infants (age 1-380 days) who received the drug by continuous intravenous infusion for analgesia before and after surgery. The leading dose and infusion rates (adjusted to age) were calculated to reach a steady-state concentration of 20 ng/ml and measured morphine concentrations (ng/ml) were effectively 20 ng/ml for age 1-7 days, but 10 ng/ml for all other ages. Morphine clearance increased steadily with age before cardiac surgery but was consistently lower (5.5-21.7 ml/min/kg) after surgery. The ratio of the morphine-6-glucuronide concentration to the morphine concentration ranged from 1.9-2.1. Infants undergoing non-cardiac surgery cleared morphine more efficiently than infants of equivalent age undergoing cardiac surgery. In addition, the clearance of morphine matured more quickly in infants undergoing non-cardiac surgery (by 1-3 months of age) than in those receiving morphine after cardiac surgery (by 6-12 months of age). Saarenmaa et al. 15 provided a rational basis for morphine administration in preterm infants in the immediate postnatal period by determining the clearance and evaluating the efficacy and adverse effects of continuous infusions. A steady-state morphine concentration was achieved between 24 and 48 hours of infusion, but morphine-6- and morphine-3-glucuronide concentrations did not reach steady state until 60 hours. Morphine clearance correlated significantly with gestational age and birth weight, and total morphine clearance and the serum concentration ratios of the metabolites to morphine correlated with gestational age and birth weight. However, the steady-state serum morphine concentration did not significantly correlate with the pain score value during the second day of life. Thus, the evaluation of pain control must be the prime guide for morphine dosing. Bouwmeester et al. 16 investigated the age-related differences in morphine requirements and metabolism in full-term neonates following major surgery. Continuous morphine infusion (10 µg/kg/h) and intermittent morphine (30 µg/kg per 3 hours) were equally effective. Younger neonates (7 days or less) had significantly higher morphine plasma concentrations and a lower ratio of morphine-6-glucuronide to morphine. Mechanically ventilated neonates (>24 hours) also had significantly higher morphine plasma concentrations. The control of pain by analgesics other than morphine has been recently reviewed elsewhere 17-19.

### Pharmacokinetics of Morphine

The 14 articles discussing the pharmacokinetics of morphine and its metabolites are summarized in Table 2. For the purposes of this discussion, they have been ordered as a function of the specific procedures on which they focused. When no procedure was specified in the report, morphine was considered to have been used as an analgesic.

### Analgesia

Hartley et al. 20 studied morphine pharmacokinetics in 17 premature neonates (gestational age 26-34 weeks) during the first day of life. Infants received either a standard or a high dose (Table 2). The kinetic parameters obtained after administration of the standard dose are also summarized in Table 2. Morphine and morphine-3-glucuronide were detected at 2 and 24 hours, whereas morphine-6-glucuronide was only detected in the 24-hour samples. The morphine-3-glucuronide plasma concentration achieved approximately 20% and 80% of the morphine values at 2 and 24 hours, respectively, whereas morphine-6-glucuronide attained 20-25% of the morphine plasma concentration by 24 hours. High plasma concentrations of morphine appeared to be well tolerated. Although the mean arterial blood pressure decreased during the first six hours of treatment, this finding was not statistically significant. Two infants experienced transient muscle rigidity, but no evidence of seizures was noted. There appeared to be no clinical advantage in using the high-dose regimen. Bhat et al. 21 studied morphine pharmacokinetics after a single dose of 100 µg/kg was administered to 20 newborn infants who were born at 26-40 weeks of gestation and who were less than 5 days of age when treated. Kinetic parameters are displayed in Table 2. Ten infants had an average gestational age and birth weight of 28 weeks and 1,020 g, respectively; seven had a gestational age and a birth weight of 33 weeks and 1,870 g, respectively; and three had a gestational age and a birth weight of 40 weeks and 3,000 g, respectively. The morphine clearance rate increased as a function of gestational age at a rate of 0.9 ml/min/kg per week of gestation. Between 18% and 22% of the drug was protein bound in the plasma. The authors concluded that there is marked variation in morphine pharmacokinetics during the neonatal period and that nearly 80% of the intravenously infused drug remains free, which might explain the high sensitivity to morphine in this age group, especially during the first week of life. Finally, Scott et al. 22 determined morphine pharmacokinetics in 48 premature neonates varying in post-conceptional age and evaluated the behavioral pain response in relationship to serum morphine concentrations. Kinetic parameters are displayed in Table 2. Morphine clearance in premature neonates increased with post-conceptional age, but no differences were observed in distribution volume among the various groups in terms of post-gestational age. Again, the behavioral pain response did not correlate with clearance or distribution levels.

### Mechanical Ventilation

Anand et al. 23 performed a multicenter study on 898 ventilated (maximum 14 days) preterm neonates (22-30 weeks gestational age) from 16 centers who were randomly assigned to placebo (n=449) or morphine (n=449). Kinetic parameters are displayed in Table 2. Clearance increased from 2.05 l/h/70 kg at 22 weeks to 6.04 l/h/70 kg at 30 weeks gestational age. The distribution volume in preterm neonates was 190 l/70 kg and did not change with age. Barret et al. 24 studied the pharmacokinetics of morphine, morphine-6-glucuronide and morphine-3-glucuronide in 19 ventilated preterm and term newborn infants. Kinetic parameters are displayed in Table 2. The patients received diamorphine loading followed by infusion over 14-149 hours. Morphine was detected in the plasma of all subjects. The clearance of morphine, but not its distribution volume, correlated with age. The morphine-3-glucuronide and morphine-6-glucuronide formation clearances were 2.5±1.8 and 0.46±0.32 ml/min/kg, respectively. Moreover, their respective excretion clearances were 0.46±0.60 and 0.71±0.36 ml/min/kg, their respective half-lives were 11.1±11.3 and 18.2±13.6 hours, and their respective distribution volumes were 0.55±1.13 and 1.03±0.88 l/kg. Thus, the metabolism of morphine in neonates was similar to that in adults, in terms of the respective contributions of each glucuronide pathway. In another study, Pokela et al. 25 reported on the pharmacokinetics of morphine in 27 term infants aged 1 week to 6 months who received a single intravenous dose of 100 µg/kg morphine after surgery (n=23) or during mechanical ventilation (n=4). Kinetic parameters are displayed in Table 2. The half-life decreased with age, with the highest value in 10 neonates younger than 1 week, a moderate value in 10 infants aged 1 week, and the lowest value in 7 infants aged from 2-6 months. The clearance of morphine increased correspondingly with age but was significantly lower in critically ill infants and it approached adult values after the age of 1 month. Chay et al. 26 evaluated morphine pharmacokinetics and pharmacodynamics after continuous intravenous infusion of morphine in 19 neonates, included preterm (n=12) and term (n=7) neonates, whose lungs were ventilated to relieve respiratory distress. The pharmacokinetic parameters are summarized in Table 2. In neonates experiencing adverse effects of morphine, the plasma clearance was found to be decreased by 50%. The morphine concentration required to produce adequate sedation was 125 ng/ml in 50% of patients, whereas concentrations above 300 ng/ml were potentially associated with adverse effects. Morphine-6-glucuronide was not detected in the plasma of any neonate, which may explain why neonates require high plasma concentrations of unchanged morphine for sedation. Additionally, the half-life, clearance and distribution volumes were not significantly different between preterm and term.

### Extracorporeal Membrane Oxygenation

Lynn and Slattery 27 studied the pharmacokinetics of morphine in 10 mechanically ventilated infants (gestational age 36-41 weeks) aged 1 day to 10 weeks when treated. Pharmacokinetic parameters are displayed in Table 2. Rates of infusion ranged from 20-100 µg/kg/h, and the duration of infusion varied from 14 hours to 15 days. Infants aged 1-4 days showed longer elimination half-lives than older infants did (6.8 *versus* 3.9 hours), and clearance in newborns was less than one third of that found in older infants (6.3 *versus* 23.8 ml/min/kg). The combination of lower clearance and a longer elimination half-life in newborns may well explain the prolonged duration of action for morphine in very young infants. Peters et al. 28 studied the pharmacokinetics of morphine in 14 neonates undergoing veno-arterial extracorporeal membrane oxygenation (ECMO). Procedures started at age <7 days and were extended for 14 days. Pharmacokinetic parameters are displayed in Table 2. Clearance at the start of ECMO was low but increased rapidly and reached normal levels after 14 days. The only covariates affecting clearance were size and age. The distribution volume increased throughout ECMO, to a volume 2.5 times greater than that in postoperative children. This finding suggests that serum concentrations decrease during the first 10 days and that dose adjustments should be performed. Clinicians should also be aware that serum morphine concentrations may decrease over time in children receiving morphine infusion while undergoing ECMO; this is attributable to increased clearance and distribution volume. Consequently, morphine therapy should be guided by clinical monitoring, preferably using validated comfort scales. Geiduschek el al. 29 determined whether the serum morphine concentration changes during the first 3 hours of ECMO and whether morphine absorption onto the membrane oxygenator is responsible for these changes. However, morphine concentrations were no different from baseline to 3 hours after beginning ECMO and no significant difference in the morphine concentration was observed in samples taken immediately proximal compared with distal to the membrane oxygenator throughout ECMO. The initiation of ECMO thus does not lead to a significant decrease in the serum morphine concentration and there is no uptake of morphine into the membrane oxygenator of the ECMO circuit. Morphine clearance for infants receiving ECMO is variable. Dagan et al. 30 studied the effect of ECMO on the pharmacokinetics of morphine in 7 infants aged 1 day to 12 months who required ECMO lasting for 40-103 hours. The reported pharmacokinetic parameters are displayed in Table 2. Morphine clearance rates doubled and serum concentrations were halved after ECMO was discontinued; consequently, higher doses of morphine were required to maintain adequate sedation. The acute decrease in the serum concentrations of morphine after cessation of ECMO were probably caused by the enhanced clearance of the drug.

### Surgery

Bouwmeester et al. 31 described the pharmacokinetics and metabolism of morphine and its metabolites in 184 children aged 3-6 years. Population parameter estimates for a one-compartment, first-order elimination model were standardized to a 70 kg body weight and are displayed in Table 2. The clearance of the morphine metabolites increased with age in parallel with glomerular filtration rate maturation. Thus, (i) morphine-3-glucuronide is the predominant metabolite of morphine in young children and (ii) the total body morphine clearance reaches 80% of adult values by 6 months. In another investigation, Barrett et al. 32 studied the pharmacokinetics of morphine in 26 premature newborns (26-38 weeks gestational age) who received a loading dose of 50 µg/kg, followed by an intravenous infusion of 15 µg/kg/h of diamorphine. The pharmacokinetic parameters are summarized in Table 2. The mean steady-state morphine plasma concentration for a diamorphine infusion rate of 15 µg/h was 62.5±22.8 ng/ml. There was a direct relationship between the gestational age of the patients and the clearance and half-life of morphine, but no relationship was found between gestational age and distribution volume. These results suggest that the currently used dosing regimen for diamorphine achieves a safe effective morphine concentration in the premature newborn but that the loading dose could be modified to achieve a more rapid onset of analgesia. Mikkelsen et al. 33 investigated the pharmacokinetics of a single dose of 15 µg/kg morphine in 5 term infants and 8 preterm infants before and after surgery. Twelve of the infants received mechanical ventilation. The median half-life in the preterm group was 9.2 hours, compared with 3.7 hours in the term infants. No significant difference was found between term and preterm in terms of distribution volume or plasma clearance. The terminal half-life decreased progressively with increasing gestational age. No correlation was found between clearance and gestational age. Finally, a larger inter-individual variation in morphine plasma concentration was observed in term infants.

### Safety and Efficacy

Farrington et al. 34 evaluated the efficacy and safety of morphine sulfate in 20 neonates requiring and recovering from surgery. Pharmacokinetic parameters are displayed in Table 2. Following surgery, each subject received an intravenous morphine loading dose (50 µg/kg), followed by continuous infusion (15 µg/kg/h) for a minimum of 24 hours. The mean percentage of unchanged morphine recovered in the urine was 39±19% of the dose administered over 12 hours. Continuous morphine therapy appears to be effective in controlling neonatal postoperative pain, as suggested by subjective observations and decreased serum β-endorphin content.

Thumel et al. 35 reported normal morphine pharmacokinetic values for healthy adults, displayed for comparison in Table 2.

## DISCUSSION

*In vivo* observations of morphine, paracetamol (acetaminophen), and propofol disposition throughout childhood confirm the overall low glucuronidation activity in neonates observed in *in vitro* studies. Compared with data related to phase 1 isoenzyme activity, data on the isoenzyme-specific phenotypic activity of uridine diphosphate glucuronosyltransferase and its covariates in neonates are limited. The present review endeavored to summarize the state of the art concerning this facet of neonatology. Stimulation of the central nervous system has been the focus of other recent reviews 36,37.

As observed in Table 1, nine of the selected articles reported on metabolic aspects of morphine processing in neonates 8-16. The most important information to be gathered from these papers is as follows: (a) sulfation is a minor component of morphine transformation 8,11; (b) glucuronidation is an essential metabolic step, converting morphine either to morphine-3-glucuronide or to morphine-6-glucuronide 9,10,12-14; (c) the relatively inactive morphine-3-glucuronide is produced as early as day 1 of life in preterm and term neonates, whereas the highly active morphine-6-glucuronide only appears on day 2 12,13; and (d) there is no obvious correlation of dose with analgesic/sedative effects during the first days of life, especially in preterm neonates.

Table 2 summarizes the pharmacokinetics of morphine, as reported in the 15 articles selected for this review 20-34. The most significant features of these reports are as follows: (a) the half-life is long in the earliest stages of life but rapidly decreases as the metabolic pathways develop 21,27,33; (b) conversely, but reflecting the same underlying characteristic, morphine clearance increases rapidly in parallel with the decrease in half-life 21-23,25,27,28,30,; and (c) no clear tendency emergeds from these studies regarding distribution volume, which appears to be independent of age.

### Other relevant aspects may be stressed here:

A. Neonates who were mechanically ventilated for longer than 24 hours had significantly higher morphine plasma concentrations than spontaneously breathing neonates did 12 and 24 hours after surgery 10,15,23-27.

B. As noted above, in premature infants who were given morphine intravenously, only morphine-3-glucuronide was detected in the plasma 2 hours after dosing, whereas morphine-6-glucuronide was detected in the plasma 24 hours after dosing. The plasma concentration ratios of morphine-3-glucuronide to morphine and morphine-6-glucuronide to morphine increased significantly with increasing birth weight 9. Morphine-6-glucuronide is a potent opioid agonist, whereas morphine-3-glucuronide functionally antagonizes several of the effects of both morphine and morphine-6-glucuronide 9. Considering these opposing properties, the proportions of the two metabolites formed are clearly relevant.

C. The pharmacokinetics of morphine vary considerably in neonates. This characteristic probably reflects immaturity of hepatic glucuronidation and altered hepatic blood flow during periods of acute illness and shunting of blood away from the liver by the ductus venosus.

D. Between 18% and 22% of morphine is bound to plasma protein in neonates 22, whereas in adults, the percentage bound to plasma protein is 35±2% 34. The greater the proportion of morphine that remains free is, the greater the proportion of morphine that could enter the neonate’s brain is, which might explain the increased sensitivity to morphine for any given dosage in neonates.

E. Estimates in adults indicate that more than 70% of morphine undergoes glucuronide conjugation and that 5-10% undergoes sulfate conjugation. In total, 3-10% of unconjugated morphine is excreted in the urine. Large variation in clearance during the first few days of life could be a reflection of immature conjugation systems or of factors related to poor hepatic blood flow.

The main limitation of the present review is the fact that only one source of information was consulted, even though a few references were manually inserted, mainly referring to the author's contribution to this field of knowledge.

In conclusion, morphine is extensively glucuronidated at positions 3 and 6 and is also sulfated at these positions. The rate of glucuronidation of morphine is lower in younger neonates compared with older infants, and the half-life and the clearance of morphine are lower in preterm infants than in term infants. Although much is already known about morphine in neonates, further research will be required to ensure that the therapeutic doses recommended for analgesia in neonates are evidence based.

## Figures and Tables

**Table 1:** Metabolic parameters of morphine in neonates. The figures are means. The adult values are normal and are used for comparison.

Reference	Number of cases	Dose	Developmental stage	Age at treatment	Procedure	Clearance (ml/min/kg)	Notes
8	16	Continuous infusion	7 Preterm 9 Term	NA	Analgesia	NA	Morphine-3 sulfate present in urine Morphine-6 sulfate absent in urine Morphine sulfation negligible in neonates
9	12	Infusion	5 Preterm 7 Term	NA	Analgesia	NA	M-3G present in all patients M-6G present in 10 patients
10	16	Average dose 92±19 µg/kg	Preterm 25-32 weeks g	<5 to 66 days (mean: 10)	Ventilation	NA	M-3G and M-6G found in two thirds of acutely ill preterm newborns
11	49	20 µg/kg/h 30 µg/kg/h	Term	1 day to 2.5 years	Post-surgery	5-21	Morphine sulfate clearance equal to adult values at age 2-25 months
12	17	100-200 µg/kg + infusion	Preterm (26-34 weeks)	1 day	Analgesia	NA	M-3G detected in plasma on day 1 M-6G detected in plasma on day 1
13	10	100 µg/kg	Preterm (25-34 weeks)	1 day	Analgesia	NA	Detected in plasma: Morphine Morphine-3-glucuronide at 2 hours Morphine-6-glucuronide at 24 hours
14	35	Dose adjusted to reach steady state: 20 ng/ml, 7-25 µg/kg/h	Term	1-7 days 8-90 days 91-180 days 180-380 days	Before and after cardiac surgery	9.2-48.9 before surgery 5.5-21.7 after surgery	M-3G detected from day 1 2 hours after dosing M-6G detected from day 2
15	31	Dose adjusted through effect	Preterm Term	10 days	Ventilation	Increase from 0.8-6.5	To provide a rational basis for morphine administration
16	68	10 µg/kg/h	Term	<7 days (52) >7 days (16)	Major surgery	NA	To determine age-related differences in morphine needs

M-3G: Morphine-3-glucuronide; M-6G: Morphine-6-glucuronide.

**Table 2:** Demographic and pharmacokinetic parameters of morphine in neonates. The figures are means. The adult values 34 are normal and are used for comparison.

Reference	Number of cases	Dose	Developmental stage	Age at treatment	Procedure	Half-life (hours)	Clearance (ml/min/kg) or (l/h/70 kg)	Distribution volume (l/kg)
20	17	100-200 µg/kg + infusion	Preterm (26-34 weeks)	Day 1	Analgesia	8.75	2.4 ml/min/kg	1.82
21	20	100 µg/kg	Preterm (10-28 weeks) Preterm (7-33 weeks) Term (3)	<5 days	Analgesia	10.0 7.4 6.7	3.4 ml/min/kg 9.6 ml/min/kg 15.5 ml/min/kg	1.84 5.18 2.9
22	48	50 µg/kg + infusion	Preterm (24-27 weeks) Preterm (28-31 weeks) Preterm (32-35 weeks) Preterm (36-39 weeks)	NA	Pain response control	6.6	2.3 ml/min/kg 3.2 ml/min/kg 4.5 ml/min/kg 7.8 ml/min/kg	2.2 2.4 2.6 3.2
23	898	100 µg/kg + infusion *versus* placebo	Preterm (22-38 weeks)	5 days to 54 weeks	Ventilation + Analgesia	NA	Increased from 2.0 l/h/70 kg to 6.0 l/h/70 kg	2.7
24	19	50 µg/kg diamorphine + infusion	Preterm (24-39 weeks) Term	1-37 days	Ventilation	11.1 (M3G) 18.2 (M6G)	4.6 ml/min/kg	0.55 (M3G) 1.03 (M6G)
25	27	100 µg/kg	Term	1 week to 6 months	Ventilation (n=4) or surgery (n=23)	8.1 to 2.6	8.7-28.0 ml/min/kg	NA
26	19	10-100 µg/kg + infusion	Preterm (12) Term (7)	NA	Ventilation for respiratory distress	9.6	2.6 ml/min/kg	2.05
27	10	20-100 µg/kg/h	Preterm Term	1-70 days	Ventilation	6.8 (1-7 days) 3.9 (older)	6.3 (1-7 days) 23.8 (older)	2.9
28	14	100 µg/kg + infusion	Term	<7 days Lasting 14 days	Membrane oxygenation	NA	2.2 l/h/70 kg, increased to 10.5 l/h/70 kg	2.0, increasing to 5.0
29	11	infusion	Term	NA	Membrane oxygenation	NA	11.7 ml/min/kg	NA
30	7	NA	Term	1 day to 12 months, lasting 40-103 hours	Membrane oxygenation	NA	9.1 during increase 17.6 post-oxygenation	NA
31	184	100 µg/kg + infusion	Term	0-3 years	Surgery	NA	71 l/h/70 kg	1.94
32	26	50 µg/kg + infusion diamorphine	Preterm (26-38 weeks)	NA	Surgery	8.9±3.3	3.6 ml/min/kg	2.7
33	13	100-200 µg/kg + infusion	Preterm (8) Term (5)	NA	Surgery	9.3 3.7	2.8 ml/min/kg 4.7 ml/min/kg	2.4 1.7
34	20	50 µg/kg + infusion	Term	NA	Surgery	6.6	2.1 ml/min/kg	5.0
35	Standard values	----	----	Adults	--------	1.9	35 ml/min/kg	3.3

M-3G: Morphine-3-glucuronide; M-6G: Morphine-6-glucuronide; NA: Not available.

## References

[1] Martin WR (1984). Pharmacology of opiates. Phamacol Rev.

[2] Stanski DR, Greenblatt DJ, Lowenstein E (1978). Kinetics of intravenous and intramuscular morphine. Clin Pharmacol Ther.

[3] Neonatal Formulary (2011). Sixth edition. John Wiley &amp; Sons, Limited European Distribution Centre New Era Estate, Oldlands Way Bognor Regis, West Sussex, PO22 9NQ, UK.

[4] Pacifici GM (2015). Clinical pharmacology of methadone in neonates and in their mothers. A review. MedicalExpress (São Paulo, online).

[5] Young TE, Mangum B (2010). Neofax, A Manual of Drugs used in neonatal care. CNS Drugs.

[6] Pacifici GM (2014). Clinical Pharmacology of Midazolam in Neonates and Children: Effect of Disease&mdash;A Review. Int J Pediatr.

[7] Pacifici GM, Clinical pharmacology of phentanyl in preterm infants (2015). A Review. Pediatrics Neonatol.

[8] Choonara I, Ekbom Y, Lindström B, Rane A (1990). Morphine sulphation in children. Br J Clin Pharmacol.

[9] Choonara I, Lawrence A, Michalkiewicz A, Bowhay A, Ratcliffe J (1992). Morphine metabolism in neonates and infants. Br J Clin Pharmacol.

[10] Bhat R, Abu-Harb M, Chari G, Gulati A (1992). Morphine metabolism in acutely ill preterm newborn infants. J Pediatr.

[11] McRorie TI, Lynn AM, Nespeca MK, Opheim KE, Slattery JT (1992). The maturation of morphine clearance and metabolism. Am J Dis Child.

[12] Hartley R, Quinn M, Green M, Levene MI (1993). Morphine glucuronidation in premature neonates. Br J Clin Pharmacol.

[13] Hartley R, Green M, Quinn MW, Rushforth JA, Levene MI (1994). Development of morphine glucuronidation in premature neonates. Biol Neonate.

[14] Lynn A, Nespeca MK, Bratton SL, Strauss SG, Shen DD (1998). Clearance of morphine in postoperative infants during intravenous infusion: the influence of age and surgery. Anesth Analg.

[15] Saarenmaa E, Neuvonen PJ, Rosenberg P, Fellman V (2000). Morphine clearance and effects in newborn infants in relation to gestational age. Clin Pharmacol Ther.

[16] Bouwmeester NJ, Hop WC, van Dijk M, Anand KJ, van den Anker JN, Tibboel D (2003). Postoperative pain in the neonate: age-related differences in morphine requirements and metabolism. Intensive Care Med.

[17] Pacifici GM (2014). Clinical pharmacology of ibuprofen in preterm infants: A meta-analysis of published data. MedicalExpress (São Paulo, online).

[18] Pacifici GM (2014). Clinical pharmacology of analgesics in infants and the pharmacologic management of pain in neonates. MedicalExpress (São Paulo, online).

[19] Pacifici GM, Allegaert K (2015). Clinical Pharmacology of Paracetamol in neonates: A review. Current Therap Res.

[20] Hartley R, Green M, Quinn M, Levene MI (1993). Pharmacokinetics of morphine infusion in premature neonates. Arch Dis Child.

[21] Bhat R, Chari G, Gulati A, Aldana O, Velamati R, Bhargava H (1990). Pharmacokinetics of a single dose of morphine in preterm infants during the first week of life. J Pediatr.

[22] Scott CS, Riggs KW, Ling EW, Fitzgerald CE, Hill ML, Grunau RV (1999). Morphine pharmacokinetics and pain assessment in premature newborns. J Pediatr.

[23] Anand KJ, Anderson BJ, Holford NH, Hall RW, Young T, Shephard B (2008). Morphine pharmacokinetics and pharmacodynamics in preterm and term neonates: secondary results from the NEOPAIN trial. Br J Anaesth.

[24] Barrett DA, Barker DP, Rutter N, Pawula M, Shaw PN (1996). Morphine, morphine-6-glucuronide and morphine-3-glucuronide pharmacokinetics in newborn infants receiving diamorphine infusions. Br J Clin Pharmacol.

[25] Pokela ML, Olkkola KT, Seppälä T, Koivisto M (1993). Age-related morphine kinetics in infants. Dev Pharmacol Ther.

[26] Chay PC, Duffy BJ, Walker JS (1992). Pharmacokinetic-pharmacodynamic relationships of morphine in neonates. Clin Pharmacol Ther.

[27] Lynn AM, Slattery JT (1987). Morphine pharmacokinetics in early infancy. Anesthesiology.

[28] Peters JW, Anderson BJ, Simons SH, Uges DR, Tibboel D (2005). Morphine pharmacokinetics during venoarterial extracorporeal membrane oxygenation in neonates. Intensive Care Med.

[29] Geiduschek JM, Lynn AM, Bratton SL, Sanders JC, Levy FH, Haberkern CM (1997). Morphine pharmacokinetics during continuous infusion of morphine sulfate for infants receiving extracorporeal membrane oxygenation. Crit Care Med.

[30] Dagan O, Klein J, Bohn D, Koren G (1994). Effects of extracorporeal membrane oxygenation on morphine pharmacokinetics in infants. Crit Care Med.

[31] Bouwmeester NJ, Anderson BJ, Tibboel D, Holford NH (2004). Developmental pharmacokinetics of morphine and its metabolites in neonates, infants and young children. Br J Anaesth.

[32] Barrett DA, Elias-Jones AC, Rutter N, Shaw PN, Davis SS (1991). Morphine kinetics after diamorphine infusion in premature neonates. Br J Clin Pharmacol.

[33] Mikkelsen S, Feilberg VL, Christensen CB, Lundstrøm KE (1994). Morphine pharmacokinetics in premature and mature newborn infants. Acta Paediatr.

[34] Farrington EA, McGuinness GA, Johnson GF, Erenberg A, Leff RD (1993). Continuous intravenous morphine infusion in postoperative newborn infants. Am J Perinatol.

[35] Thummel KE, Shen DD, Isoherran N, Brunton L, Chamber B, Knollman E (2011). Dosing and optimization of dosage regimens. In Goodman & Gilmans The Parmacological Basis of Therapeutics.

[36] Pacifici GM (2014). Clinical Pharmacology of Caffeine Citrate in Preterm Infants. MedicalExpress (São Paulo, online).

[37] Pacifici GM (2014). Clinical Pharmacology of Theophylline in Preterm Infants: Effects, Metabolism and Pharmacokinetics. Current pediatr reports.

